# Weaning Holstein Calves at 17 Weeks of Age Enables Smooth Transition from Liquid to Solid Feed

**DOI:** 10.3390/ani9121132

**Published:** 2019-12-12

**Authors:** Sarah Schwarzkopf, Asako Kinoshita, Jeannette Kluess, Susanne Kersten, Ulrich Meyer, Korinna Huber, Sven Dänicke, Jana Frahm

**Affiliations:** 1 Institute of Animal Science, Faculty of Agricultural Sciences, University of Hohenheim, 70599 Stuttgart, Germany; Sarah.Schwarzkopf@uni-hohenheim.de (S.S.); asakokinoshita@googlemail.com (A.K.); Korinna.Huber@uni-hohenheim.de (K.H.); 2 Institute of Animal Nutrition, Friedrich-Loeffler-Institute, 38116 Braunschweig, Germany; jeannette.kluess@fli.de (J.K.); Susanne.Kersten@fli.de (S.K.); Ulrich.Meyer@fli.de (U.M.); Sven.Daenicke@fli.de (S.D.)

**Keywords:** weaning age, Holstein calves, growth, milk replacer, metabolism, development

## Abstract

**Simple Summary:**

Weaning calves from liquid to solid feed can be a stressful event in their life and can affect growth, development and welfare. It is commonly done at the age of 7 to 8 weeks on dairy farms, but weaning at a greater age could potentially reduce the associated stress. Therefore, it might improve growth rates and enable a smooth transition to an adult liver metabolism. To confirm this hypothesis this study evaluated the effect of two different weaning ages (7 vs. 17 weeks of age) on female Holstein calves. Furthermore, the effect of mothers’ parity was analyzed (primiparous vs. multiparouos). Primiparous cows were often immature and still developing during their first pregnancy. This can lead to negative intrauterine conditions and result in long-term changes in the calf’s metabolism. Late-weaned calves consumed high amounts of concentrate feed before weaning despite their high milk replacer intake, indicating the maturation of their rumen. In addition, they experienced a smooth transition to an adult liver metabolism as reflected by steady plasma glucose and cholesterol concentrations. Later weaning corrected the reduced growth of calves born to primiparous cows as well, indicating that those particularly benefitted from late weaning. All benefits were indicated by slower changes of blood metabolites and higher growth rates, which might lead to better health and productivity in their subsequent lifetime.

**Abstract:**

Development of calves depends on prenatal and postnatal conditions. Primiparous cows were still maturing during pregnancy, which can lead to negative intrauterine conditions and affect the calf’s metabolism. It is hypothesized that weaning calves at higher maturity has positive effects due to reduced metabolic stress. We aimed to evaluate effects of mothers’ parity and calves’ weaning age on growth performance and blood metabolites. Fifty-nine female Holstein calves (38.8 ± 5.3 kg birth weight, about 8 days old) were used in a 2 × 2 factorial experiment with factors weaning age (7 vs. 17 weeks) and parity of mother (primiparous vs. multiparous cows). Calves were randomly assigned one of these four groups. Live weight, live weight gain and morphometry increased over time and were greater in calves weaned later. Metabolic indicators except total protein were interactively affected by time and weaning age. Leptin remained low in early-weaned calves born to primiparous cows, while it increased in the other groups. The results suggest that weaning more mature calves has a positive effect on body growth, and calves born to primiparous cows particularly benefit from this weaning regimen. It also enables a smooth transition from liquid to solid feed, which might reduce the associated stress of weaning.

## 1. Introduction

Calves are born as functional monogastric animals that rely on nutrients from milk or milk replacer (MR) [1,2]. Therefore, weaning is a vital event in the young ruminant’s life, as it means that lactose and milk fat are no longer available as main sources for energy metabolism. The change from functional monogastric to ruminant not only relies on volatile fatty acids (VFA) production in the rumen to supply energy, but also on well-functioning endocrine and biochemical features such as ruminant-specific insulin homeostasis and hepatic gluconeogenesis. Thus, weaning causes stress [3,4], and could affect animal welfare, growth, development and future performance [5,6,7]. Calf mortality was high (5%–9%) in the last decades [8,9]. For economic reasons the preweaning period was substantially shortened in dairy cow production systems [10]. In the USA and Canada, the typical weaning age was 6–8 weeks [9,11]. Early weaning was introduced to promote the early intake of concentrate feed and hay, which are cheaper feeds than milk or MR. Therefore, feeding strategies for dairy calves focused on increasing the capacity for solid feed and accelerating the development of the forestomach system. In scientific studies, only a few variables such as beta-hydroxybutyrate in the plasma and rumen epithelium growth are used to indicate this [1,2]. A suggested potential benefit of early weaning was a faster rumen development [12], whereas the maturity of other parts of the gastrointestinal tract and organs like the liver was not considered. Therefore, little research was done about weaning calves older than 14 weeks [13].

Weaning calves which are more mature was discussed to have many benefits for the growth and development of dairy calves. It has been associated with greater live weight gain (LWG) and improved gastrointestinal development at the time of weaning [5]. For the first 56 days of life, feed efficiency (gain: feed ratio) tended to be greater for calves that were fed milk compared to grain [14]. The utilization of solid feed (corn silage, wheat straw, concentrate) for growth increased until 27 weeks of age [15].

Considering the great impact of weaning on dairy calves, it is crucial to find an optimal age for it. In this study, the optimal age denotes sufficient maturity in all organ and tissue functions, and not only in the ruminal digestion of solid feed. In fact, we define maturity for weaning as the capacity of all organs to fulfill the digestive and metabolic needs for changing to a ruminant status.

In the present study, early weaning was conducted at the age of 7 weeks, as this is a common management decision taken on dairy farms [9,11]. As opposed to that, late weaning was executed at the age of 17 weeks, because the reticulorumen volume of calves reaches adult proportions of 23 to 36 L/100 kg of ingesta-free body weight at 12 to 16 weeks [16].

The prenatal period as well as the early postnatal period are critical stages of development at which metabolic imprinting may occur and have great impact on health and performance in adult life [17]. Opsomer et al. [18] concluded in their review article that the parity of the mother could have a major impact, as older cows are lactating and heifers are still growing during pregnancy. Older cows tended to give birth to larger calves [19]. As birth weight was associated with improved glucose metabolism in humans in adulthood [20], mother’s parity can influence the calf in the long-term. In most studies on calf development, the authors did not consider this parity of the mother as a potential influencing factor of development. Furthermore, few studies examined post-weaning development in female calves for a longer period.

The present study aimed to determine the influence of mother’s parity and calves’ weaning age on growth, energy and protein metabolism and on endocrine regulators. Growth performance and weight gain was evaluated by morphometric measures, and metabolic maturity was assessed by insulin, leptin and adiponectin as regulators of organ maturation. Since energy and protein metabolism are crucial for growth and development, indicators such as glucose, beta-hydroxybutyrate (BHB), non-esterified fatty acids (NEFA), cholesterol, urea and total protein were measured by spectrophotometric methods.

To assess metabolic imprinting as well as economic aspects, these animals were monitored in an ongoing observational study.

## 2. Materials and Methods

In accordance with the German Animal Welfare Act, pertaining to the protection of experimental animals and approved by The Lower Saxony State Office for Consumer Protection and Food Safety (LAVES), Oldenburg, Germany, the present trial was carried out at the experimental station of the Institute of Animal Nutrition, Friedrich-Loeffler-Institute (FLI), Brunswick, Germany (file No.: 33.19-42502-04-15/1858).

### 2.1. Animals, Housing, and Diets

Female German Holstein calves (*n =* 59) were studied from day of birth until day 149 ± 2 (mean ± standard deviation (SD)) of life. All calves originated from one established herd of Holstein cows and were born within a seasonal calving period of three months (October–December). They were all vaccinated with inactive *Mannheimia haemolytica* serotype A1 and A6, parainfluenza3 vaccine and bovine respiratory syncytial virus (Bovigrip^®^ RSP plus, MSD Animal Health, Unterschleißheim, Germany) in weeks 5 and 9 of age, against *Trichophyton* in weeks 6 and 8 of age with live attenuate vaccine (Bovilis^®^ Ringvac, MSD Animal Health, Unterschleißheim, Germany) and against blue tongue disease (BTV) (Zulvac 8, Zoetis Belgium SA, Louvain-la-Neuve, Belgium) in weeks 11 and 15 of age.

Calves were weighed with an electronic scale directly after birth and received 3 L of colostrum through a nipple bucket within 2 h after birth. The quality of colostrum was evaluated using a colostrum densimeter (Wahl GmbH, Dietmannsried, Germany) and had to be greater than 1035 g/L, otherwise colostrum from another cow from the same herd was used. They were moved 2–3 h after birth into straw-bedded single hutches and were fed twice with three liters of pooled herd milk each day. The pre-experimental feeding period for neonatal calves was done according to standard dairy management practice at the experimental station. In detail, starting at the age of three days, milk replacer (MR) (NOLAC GmbH, Zeven, Germany, Table 1) was mixed with the pooled herd milk, with gradually increasing amounts from 0.3 kg MR powder/d (third day of life) to 0.9 kg MR/d (fifth day of life), while the maximum of 6 L liquid feed with a concentration of 150 g/L MR was available (Table 2). Calves entered the study at a mean age of 8 ± 1.9 days and 44.5 ± 5.2 kg of live weight and moved into straw-bedded stables with MR and concentrate self-feeding systems (Förster-Technik GmbH, Engen, Baden-Württemberg, Germany). Differential feeding and monitoring of feed intake were achieved using a transponder in the calf’s ear. They were randomly allocated to either early weaning at 7 weeks of age (early-weaned calves from multiparous cows (earlyMC)/early-weaned calves from primiparous cows (earlyPC)) or late weaning at 17 weeks of age (late-weaned calves from multiparous cows (lateMC)/late-weaned calves from primiparous cows (latePC)) group considering an equal allocation of calves from primiparous cows (PC) and calves from multiparous cows (MC). Our experimental trial started with 0.9 kg MR powder/d, which were available for all calves for the first five experimental days. MR was increased gradually within the next five days (experimental days 6 to 10) from 0.9 kg MR powder/d to 1.35 kg MR powder/d, and remained at this level until the beginning of the weaning period (early-weaned group = day 28, late-weaned group = day 98). Concentration of MR was continuously at 150 g MR powder/L over the complete experimental time, and a maximum of 9 L liquid feed was available (Table 2). Over the entire trial, all calves received hay and water ad libitum and had access to a maximum of 2 kg concentrate feed per day until weaning. With the start of weaning at experimental day 98, the amount of concentrate feed was reduced to 1 kg/d according to standardized dairy management practice at the experimental station. During weaning, the milk replacer was gradually reduced within 14 days from 1.35 kg/d to 0.3 kg/d. Post-weaning calves were moved to another barn and received hay and a total mixed ration (TMR) consisting of 48% grass, 32% maize silage and 20% concentrate feed.

The ingredients of MR powder and concentrate feed are shown in Table 1. Composition of concentrate feed, roughage, milk replacer and TMR were determined according to the suggestions of the Association of German Agricultural Analysis and Research Centers [21] (Table 3).

### 2.2. Morphometry of Calves

Concerning morphometry, the hip height, withers height, back length, body length and heart girth were determined as shown in Table 4 at days 1, 7, 14, 28, 42, 56, 70, 84, 98, 112, 126 and 140 of this trial. Hip and withers height were measured with a folding rule, the other measurements were taken with a measuring tape. Live weight (LW) was recorded on day of birth, and also on days 1, 28, 42, 70, 98, 112 and 140 with an electronic scale. Live weight gain (LWG) in kg per day was calculated from this data by dividing the weight gain between our sample days through the number of days between sampling.

### 2.3. Collection and Analysis of Blood Samples

Blood samples of each individual animal were taken on experimental days 1, 28, 42, 70, 98, 112 and 140 by jugular venipuncture and collected in serum and ethylenediaminetetraacetic acid (EDTA) plasma tubes (10 mL tubes; Sarstedt, Nuembrecht, Germany). Serum tubes were incubated for 30 min at 30 °C. After centrifugation at 3000× *g* for 15 min at 15 °C, serum and plasma aliquots were stored at −80 °C for subsequent analyses. Serum leptin concentrations were determined using a competitive enzyme immunoassay according to Sauerwein et al. [22]. Adiponectin concentrations were analyzed in serum with an indirect competitive bovine specific enzyme-linked immunosorbent assay (ELISA) according to Mielenz et al. [23]. Analyses of serum concentrations of beta-hydroxybutyrate (BHB), non-esterified fatty acids (NEFA), cholesterol, urea, total protein and glucose were done by an automatic analyzing system, based on spectrometric measures (Eurolyser, Type VET CCA, Salzburg, Austria). Insulin concentration in plasma was analyzed with a bovine insulin ELISA (Mercodia, Sweden).

### 2.4. Statistical Analysis

Live weight (LW), live weight gain (LWG), hip and withers height, body length, heart girth, back length, serum glucose, beta-hydroxybutyrate (BHB), non-esterified fatty acids (NEFA), leptin concentrations and plasma insulin concentrations were presented as least squares means (LSMeans) and standard errors (SEs) which were evaluated by repeated measures using the PROC MIXED procedure in SAS (V 9.4., SAS Institute Inc., Cary, NC, USA), and employing a restricted maximum likelihood model (REML). The model included a fixed factor of time, weaning age, parity of the mother and their interactions while the time was taken into consideration by a “REPEATED” statement. Best fitting covariance structures (compound symmetry, autoregressive and unstructured) was tested and used, based on the Akaike Information Criterion (AICC). Significant effects were further tested with the Tukey–Kramer procedure using the piecewise differentiable (PDIFF) procedure. Visualization and correlations computed as Pearson correlation coefficients were done using GraphPad Prism 6.0 (GraphPad software, San Diego, CA, USA). For all statistical tests, *p* < 0.05 was the level of significance. For visualization, the measurements on serial time points were interpolated linearly.

## 3. Results

Multiparous cows were 1592 ± 805 days (Mean ± SD) old when they gave birth to MC. Their mean lactation number was 1.875 ± 0.074 lactations (Mean ± SD). The age of primiparous cows was 710 ± 67 days (Mean ± SD) at calving. Birth weight of PC was 37.9 ± 4 kg (Mean ± SD) and birth weight of MC was 39.6 ± 6 kg (Mean ± SD).

### 3.1. Feed Intake

**Figure 1:**
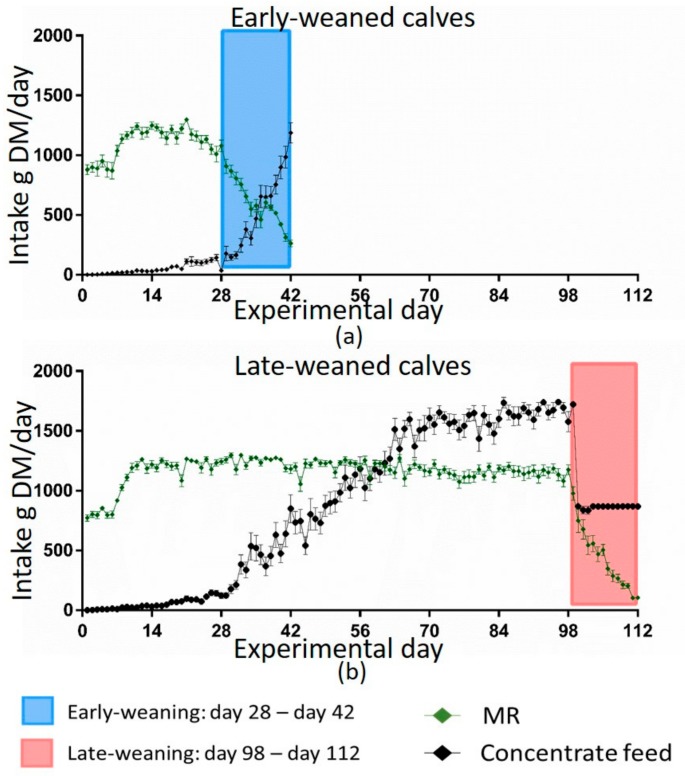
Milk replacer (MR) and concentrate feed intake in g dry matter/d for early-weaned calves (**a**) and late-weaned calves (**b**). Early-weaned calves (*n =* 24) were weaned gradually between days 28 and 42 of trial. Late-weaned calves (*n =* 28) were weaned gradually between days 98 and 112 of trial. Amount of concentrate feed was limited to 1 kg/d after weaning for early-weaned calves and during weaning period of late-weaned calves. Data shown as means ± the standard error of the mean (SEM) evaluated with GraphPad Prism 6. Due to technical problems, not all calves could be included in monitoring feed intake.

There was no difference in feed intake between calves from multiparous (MC) and primiparous cows (PC) in both weaning groups. Therefore, all data from calves of one weaning group were combined for the visualization of feed intake patterns in early and late weaned calves (Figure 1). Both groups had the same MR intake for the first 28 days of trial. Early-weaned calves consumed 11,288 g MR DM on average during their weaning period (days 28–42) whereas late-weaned calves consumed 7182 g MR DM on average during their weaning (days 98–112). Thus, the MR intake during weaning was lower for late-weaned calves compared to early-weaned calves. Early-weaned calves consumed their whole MR allowance until weaning, whereas late-weaned calves reduced their MR intake earlier than they had to. Both weaning groups started to consume concentrate feed around day 21 of trial. Early-weaned calves increased their concentrate feed intake during their weaning period. Late-weaned calves also increased their intake until day 63 of the trial and then consumed between 1500 and 1700 g concentrate DM/day until weaning. Late-weaned calves increased their roughage intake when the MR supply was reduced, whereas early-weaned calves started to consume roughage when weaning was already done (data not shown).

### 3.2. Morphometry

**Figure 2:**
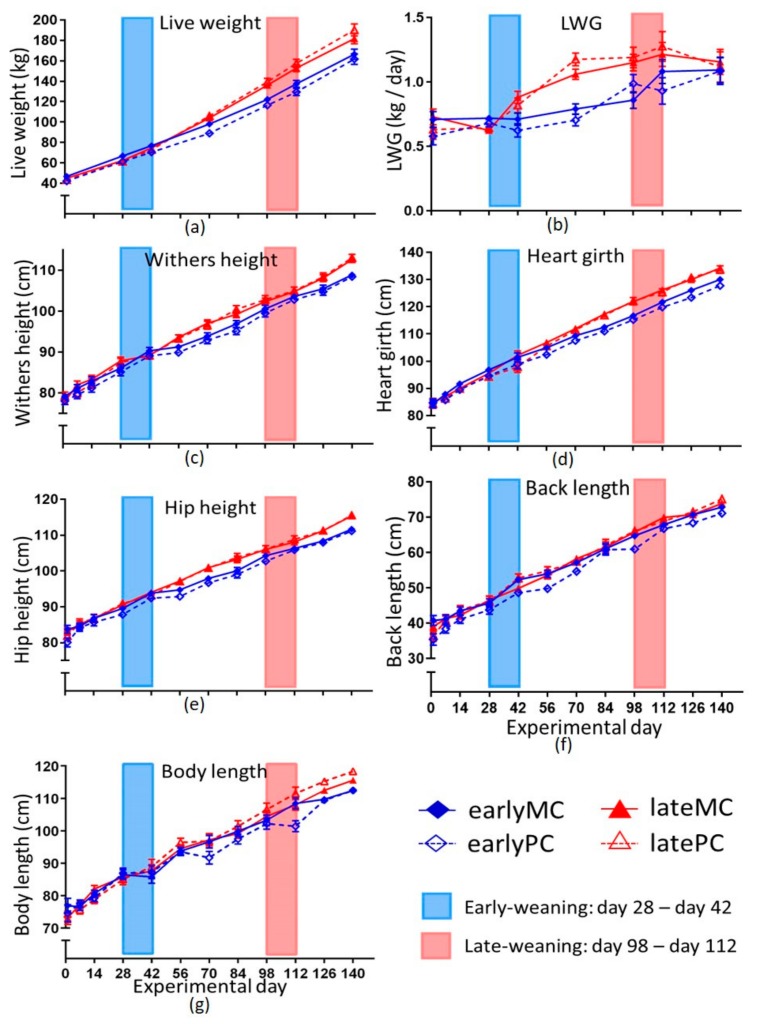
Morphometry of calves. Shown are live weight (**a**), live weight gain (LWG) (**b**), withers (**c**), heart girth (**d**), hip height (**e**) and back length (**f**). Early-weaned calves were weaned gradually between days 28 and 42 of the trial. Late-weaned calves were weaned gradually between days 98 and 112 of the trial. Data shown as LSmeans ± SEM, early-weaned calves from multiparous cows (earlyMC) *n =* 16, late-weaned calves from multiparous cows (lateMC) *n =* 16, early-weaned calves from primiparous cows (earlyPC) *n =* 15, late-weaned calves from primiparous cows (latePC) *n =* 12.

Morphometric variables increased over time (*p* < 0.001) and were greater in late-weaned calves. Interactions between time and weaning group were also observed to be highly significant for LW, withers and hip height (*p* < 0.001), heart girth (*p* < 0.01) and body length (*p* < 0.05; Figure 2). LW was greater for all calves in the late-weaned group from day 70 until the end of trial (Figure 2a). On day 140 the mean live weight of early-weaned calves was 164.1 kg ± 3.65 kg, whereas that of late-weaned calves was 186.1 ± 3.88 kg (*p* = 0.009). LWG (Figure 2b) was strongly influenced by the weaning age (*p* < 0.001). Additionally, a significant interaction between time and weaning age was found (*p* < 0.001). Late-weaned calves had a significantly higher LWG from day 42 until day 98 of our trial (*p* < 0.05). Withers height differed significantly between late- and early-weaned calves on day 84 (*p* = 0.014) and day 140 (*p* < 0.001). Hip height differed significantly between the weaning groups from day 56 until day 84 (*p* < 0.05) and on day 140 (*p* < 0.001; Figure 2c,d). Late-weaned calves had a greater heart girth from day 84 onwards (*p* < 0.05; Figure 2f). Body length was significantly lower for early-weaned calves at the end of trial on day 126 and day 140 (*p* < 0.01). Back length was the only morphometric variable influenced by parity of the mother as indicated by an interaction between parity and weaning age (*p* = 0.011). EarlyPC had significantly lower back length than earlyMC (*p* < 0.001), whereas it did not differ in the parity groups of late-weaned calves (Figure 2e).

### 3.3. Blood Parameters

**Figure 3:**
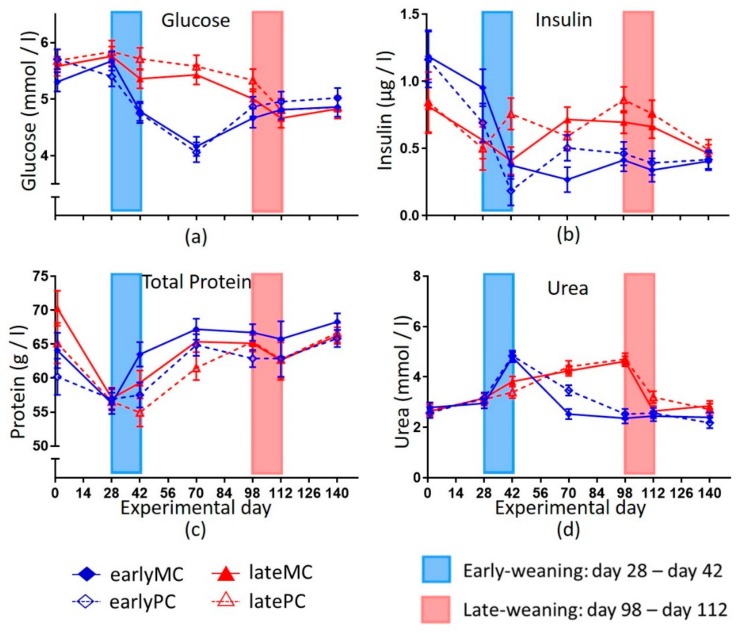
Blood concentrations of the glucose (**a**), insulin (**b**), total protein (**c**) and urea (**d**) of calves. Early-weaned calves were weaned gradually between days 28 and 42 of the trial. Late-weaned calves were weaned gradually between days 98 and 112 of this trial. Data shown as LSmeans ± SEM, early-weaned calves from multiparous cows (earlyMC) *n =* 16, late-weaned calves from multiparous cows (lateMC) *n =* 16, early-weaned calves from primiparous cows (earlyPC) *n =* 15, late-weaned calves from primiparous cows (latePC) *n =* 12.

**Figure 4:**
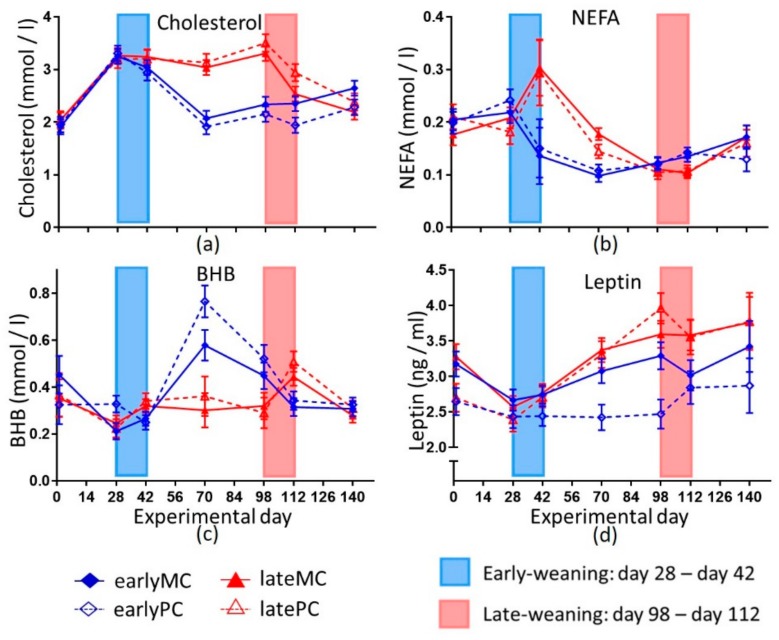
Indicators for lipid metabolism of calves. Shown are cholesterol (**a**), non-esterified fatty acids (NEFA) (**b**), beta-hydroxybutyrate (BHB) (**c**) and leptin (**d**). Early-weaned calves were weaned gradually between days 28 and 42 of this trial. Late-weaned calves were weaned gradually between days 98 and 112 of this same trial. Data shown as LSmeans ± SEM, early-weaned calves from multiparous cows (earlyMC) *n =* 16, late-weaned calves from multiparous cows (lateMC) *n =* 16, early-weaned calves from primiparous cows (earlyPC) *n =* 15, late-weaned calves from primiparous cows (latePC) *n =* 12.

Time had a significant effect on all measured variables in the blood (*p* < 0.001) and there was an interaction of time and weaning age observed for all variables except total protein (Figure 3 and Figure 4). On day 70, which was between the two weaning periods, the two weaning groups differed highly significant in their serum glucose concentration (*p* < 0.001, Figure 3a). Blood glucose concentration increased significantly from day 70 to day 112 in the early-weaned calves (*p* = 0.014). Insulin concentration dropped on days 98 and 112 in early-weaned calves and stayed below those insulin concentrations of late-weaned calves during the rest of the trial until day 140 (Figure 3b). Total protein concentration in calves (Figure 3c) was influenced by mother’s parity (*p* = 0.021) and was higher in MCs. Urea concentration (Figure 3d) in late-weaned calves increased constantly up to day 98 and started to drop to the initial level during weaning. In early-weaned calves, it increased during weaning and decreased afterwards. Therefore, they reached lower urea concentrations after weaning than late-weaned calves from day 42 until day 98 (*p* < 0.001).

Cholesterol concentrations increased similarly in all groups from day 1 to day 28. After weaning, it decreased in the early-weaned calves from day 28 to day 70 (*p* < 0.001). Therefore, they showed lower cholesterol concentrations than late-weaned calves until day 112 (*p* < 0.01). From day 70 to day 140 it increased significantly in early-weaned calves (*p* = 0.011; Figure 4a). NEFA concentrations decreased with weaning in early-weaned calves. Therefore, late-weaned calves had higher NEFA concentrations on day 70 (*p* = 0.001; Figure 4b). The serum BHB concentration increased after weaning for the early-weaned calves (day 42–70, *p* < 0.001), whereas it remained low in the late-weaned calves and increased after their weaning period (day 112–140; *p* < 0.001). As a result, a significant difference in serum BHB concentration was observed between the weaning groups on day 70 (*p* = 0.001; Figure 4c). After day 70, BHB concentration decreased until day 140 in early-weaned calves (*p* < 0.001). BHB concentration was negatively correlated with glucose concentration when all treatments and time points were considered collectively (*p* = 0.0001; r = −0.1895). Serum leptin concentration showed a significant interaction of weaning age and time (*p* < 0.001), and was also influenced by parity of the mother (*p* < 0.05). Serum leptin concentration increased from day 28 to day 140 in late-weaned calves (*p* = 0.008), whereas no significant increase was found in early-weaned calves (Figure 4d). Calves’ plasma leptin concentrations correlated positively with the lactation number of the mother (*p* = 0.0015; r = 0.4177; data not shown). All measured blood metabolites except insulin (Figure 3a), total protein (Figure 3c) and NEFA (Figure 4b) were affected by weaning age. Weaning age did not affect Adiponectin concentrations, but there were significant effects of time (*p* < 0.001) and interaction between time and parity (*p* = 0.031; data not shown).

## 4. Discussion

This study assessed the impact of two different weaning ages. Furthermore, calves were grouped according to their mother’s parity. Precisely, calves born to primiparous and born to multiparous cows were allocated to both weaning groups. To assess the effect of weaning age, a high-quality MR was used, consisting mostly of milk components (Table 2). One liter of MR contained the same amount of protein as whole milk (36.13 g XP/L MR vs. 35 g XP/L whole milk). The amount of MR powder used in literature ranges from 0.383 kg per day to 1.49 kg per day [24], thus the calves in the present study received a high amount of MR, which was quite similar to an ad libitum intake [25]. Voluntary DM intake/day from MR was lower than 1300 g/day in the first 8 weeks of life [26], which was the maximum allowance in the current study. Therefore, the effects of weaning age were assessed under sufficient milk-derived energy and nutrient supply, and not negatively influenced by a low amount and quality of MR.

### 4.1. Feed Intake

Data from computer-controlled mangers for roughage were not shown as there were technical problems with recognizing the individual calf, precluding data collection. As expected, MR intake was not different in the four groups during the first 28 days of trial when MR allowance was the same for all calves. Afterwards, MR intake followed the regimen of weaning. However, the pattern of voluntary solid feed intake over time varied between the weaning groups. Late-weaned calves consumed concentrate feed—despite consumption of the full amount of MR and even before MR supply was reduced, which led to a greater concentrate feed intake at the beginning of their weaning compared to the early-weaned group. This was in line with previous findings [5,13]. Later weaning (12.7 weeks) resulted in concentrate feed intake before weaning (close to 0.5 kg/d), whereas early-weaned calves (6.7 weeks) did not increase their concentrate intake before weaning started [13]. Eckert et al. [5] observed the same feed intake pattern for calves that were weaned with 6 or 8 weeks, with calves weaned later consuming more concentrate feed 1 week pre- and post-weaning. Male calves permitted to choose their preferred feed between MR and different solid feed components started to consume concentrate feed at the age of 49 days [26]. Despite the high MR allowance, calves consumed solid feed as observed in the present study. As milk production in the first lactation was associated with a higher intake of grain and forage at weaning [7], voluntary solid feed intake before weaning can be a potential benefit for later life production. Higher starter intake was related to a higher weight gain during weaning [13]. Late-weaned calves probably consumed more energy until their weaning, as they had a higher concentrate feed intake and still consumed MR. This was associated with the higher growth rates of late-weaned calves (Figure 2). This also indicates that even though the late-weaned calves had a high MR supply for 15 weeks, they started to consume solid feed and their rumen probably started to develop and to maturate. They even restricted their MR intake during the weaning period voluntarily more than they had to, which might indicate the ability of mature organs to function ruminant-specifically. 

It is proposed that solid feed was digested in the rumen as indicated by several rumen development parameters (Schwarzkopf et al., unpublished data). This indicated a development of rumen digestive functions despite high MR intake. Besides gastrointestinal development, however, liquid feeding in addition to voluntary solid feed intake over 17 weeks in the early life of calves might also be of advantage for other body functions and endocrine regulatory processes, as demonstrated in the following sections.

### 4.2. Morphometry of Calves

Weaning calves at a more mature developmental stage (17 weeks of life) resulted in increased LWG and higher LW (Figure 2). This was also demonstrated by several other studies [5,27,28]. The reticulorumen reached its adult proportions at the age of 12 to 16 weeks [16]. Additionally, the utilization of solid feed for body growth increased with age. Berends et al. [15] adjusted the quantity of MR for male calves to achieve the same weight gain across different solid feed levels. This way, they measured an increasing utilization of solid feed until the age of 27 weeks. If this was a sign of maturity and a proper function, it could be an explanation why later-weaned calves had a greater live weight and were able to maintain this at least 4 weeks after weaning. It could mean that the early-weaned young ruminants were physiologically unable to use all the energy provided with solid feed, and their preferred sources of energy remained lactose and milk fat. As promoted by early-weaning, the intake of solid feed instead of MR also resulted in a greater need of energy for ruminal activity. Elevated muscle work in the rumen needs energy. Furthermore, heat production through fermentation in the rumen [29] increased the energetic need for thermoregulation. Thus, the metabolic rate for maintenance might increase by solid feed intake. This could also be an explanation for the lower LWG in early-weaned calves compared to the late-weaned groups. Early-weaned calves were unable to compensate the reduced growth and could not catch up with weight, wither and hip height, body length and heart girth at least until the age of 5 months (Figure 2, *p* < 0.05). Furthermore, back length showed a significant interaction of mother’s parity and weaning age (*p* = 0.011), showing shorter back lengths in earlyPC. Reduced back length is a well-known symptom of prenatal imprinting by intrauterine malnutrition in rodents, sheep and humans [30]. Weaning late, however, was advantageous to correct the imprinted change in body proportions, as latePC did not express shorter back lengths.

### 4.3. Blood Metabolites

Collection of blood samples was always in the morning between eight and ten, but it was not controlled for feed intake. Part of the variance of serum insulin and glucose concentration could be a result of different times of feed intake relative to sampling. In milk-fed calves, blood glucose increased 1 h after feeding and then decreased rapidly during the next 2 h [28]. A high capacity for hepatic gluconeogenesis is an essential metabolic feature for a ruminant due to low intestinal glucose availability. Weaning late at 17 weeks of age resulted in a smooth transition of glucose metabolism from lactose to endogenous glucose production by the liver without any signs of dysregulation. In early-weaned calves, weaning led to a strong decrease in blood glucose concentration (Figure 3a), as lactose is the most important source of glucose for young calves. Obviously, early-weaned calves were not able to compensate the lack of dietary glucose (lactose) by hepatic gluconeogenesis. The glucose gap was closed slowly. There was a significant increase in blood glucose concentration from day 70 to day 112 in the early-weaned calves (*p* = 0.014), which indicated that liver gluconeogenetic function maturated slowly until 4 months of age. This is in accordance with findings of other authors [27]. Calves that were weaned at the age of 5 weeks had lower blood glucose concentrations than calves that still received MR, even 7 weeks after weaning [28].

Low blood glucose concentrations are detrimental for a developing young animal, since related endocrine status is concomitantly changed. The decrease in glucose concentrations at early-weaning resulted in a decrease in serum insulin concentrations. In more mature calves, the endocrine system was smoothly adapting when weaning was done with only marginal changes in hormone concentrations. The decrease of insulin was steeper for early-weaned calves and resulted in more abrupt changes (Figure 3b). Therefore, weaning stress of calves was attenuated when weaned later, because it has more time to mature and adapt. In accordance, other studies also showed that a lower intake of MR decreased insulin and insulin like growth factor 1 (IGF1) concentrations in calves [24,31,32]. Therefore, a catabolic status was most likely established in early-weaned calves, as insulin and IGF1 are the strongest anabolic hormones.

Consequently, lipolysis and proteolysis most likely were promoted to gain energy and produce precursors for gluconeogenesis. The catabolic state, however, was not able to increase glucose concentrations for several weeks in early-weaned calves. Both pathways led to an increase in ketone bodies in blood when the oxidative capacity of mitochondria was limited [33]. The negative correlation between glucose and BHB concentrations supported the hypothesis that BHB originated from lipolysis and proteolysis because of glucose shortage. NEFA derived from lipolysis were used in beta-oxidation. Hence, the blood concentrations decreased in early-weaned calves (Figure 4b). Simultaneously, the blood BHB concentration increased, reflecting a low capacity of hepatic oxidative phosphorylation. Furthermore, an incomplete oxidation of amino acids could also result in a higher BHB concentration. The decrease in cholesterol concentrations might also be linked to higher ketone body production, as the precursor metabolite (3-Hydroxy-3-methyl-glutaryl-CoA) for cholesterol production was used for BHB production [34]. Low insulin concentration led to a lower activation of HMG-CoA-reductase, which is vital for cholesterol biosynthesis [35]. This might also be a reason for lower cholesterol production. There was a significant increase in cholesterol concentration in the early-weaned groups from day 70 to day 140 of this trial (*p* = 0.011), which reflected that the liver was unable to produce as much cholesterol in the young, early-weaned calves on account of later production when they were more mature. A higher energy supply enhanced cholesterol biosynthesis in 16-week-old bull calves [36], which is in line with our findings in late-weaned female calves that received more energy through MR and a higher concentrate feed intake (Figure 1). Moreover, the MR is a nutritional source for cholesterol, which is no longer available after weaning. Therefore, late-weaned calves had a constant supply of cholesterol until day 98 of the trial. The liver of early-weaned calves was not able to produce enough cholesterol and glucose to compensate the dietary lack through weaning for several weeks. Possibly, these are not the only metabolic pathways that did not mature in early-weaned calves, and other important ones were impaired, as well.

Blood BHB concentration was used as a marker for rumen development as it originated from rumen wall ketogenesis [37]. Many authors observed a rise in blood BHB at weaning and with starter intake [14,38]; while lower concentrations were detected in ad libitum MR fed calves (0.14 ± 0.01 mmol/L) compared to calves fed with restricted MR (0.17 ± 0.01 mmol/L) [31]. A similar pattern was observed in this study, as serum BHB concentrations were higher in early-weaned calves compared to late-weaned calves still on MR feeding on day 70 (Figure 4c). But these higher concentrations declined again with age (*p* < 0.001). Thus, their relevance as a marker for rumen development could be questioned. It is likely that to some extent BHB was derived from an incomplete oxidation of nutrients, such as fatty acids and amino acids as described above. Parts of higher NEFA concentration in late-weaned calves on day 70 might also be explained by NEFA content in MR (Figure 4). Other authors had also seen that weaning resulted in lower NEFA concentration compared to calves that received MR [14,39]. Low glucose and high BHB after weaning indicated that capacity of liver functions was less developed in early-weaned calves. The increase in plasma urea concentration during weaning could have resulted from elevated proteolysis as well. After weaning the urea concentration likely decreased because the rumen used it in the ruminohepatic recycling of nitrogen. Another explanation might be a lack of microbial protein and low protein sustenance. Hence, plasma urea concentrations were lower in early-weaned calves than in late-weaned calves that still received MR and thus got enough protein for body protein turn-over.

#### Influence of Mother’s Parity on Early- and Late-weaned Calves

As discussed before, heifers have to allocate nutrients and energy between their own body needs and the requirements of the fetus. This could create unfavorable conditions such as intrauterine malnutrition for the unborn calf, which could affect them for their whole life [18]. Besides reduced back length, a lower leptin concentration appeared to be another sign of intrauterine imprinting by malnutrition (Figure 4d). Furthermore, the lactation number of the dams and serum leptin concentrations in their 1-week old calves were positively correlated (*p* = 0.0015; r = 0.4177). Leptin plays an important role in the onset of puberty and regulation of the immune system [40,41]. 

Low leptin concentration in early postnatal life was associated with a leptin resistance in later life in rats [42]. Thus, the lower leptin concentrations in PC could indicate a potential risk factor for a dysregulated energy metabolism and development also in later life [43]. The main effect of leptin is a decrease in feed intake. In general, leptin concentration changes during a long-term negative energy balance in mammalians [44]. Hence, the lower serum leptin concentrations in the PC can be hypothesized as a sign of hunger and lack of adipose deposition.

### 4.4. Implications and Perspectives

Most of the evaluated blood metabolites did not differ among the groups at the age of 5 months, but changes occurred more abruptly for early-weaned, and slower for later-weaned calves. Hence, early weaning may cause more postnatal metabolic stress. This experience could also lead to metabolic imprinting and affect health and productivity in later life [20]. Kenéz et al. [6] examined a reduced amount of MR supply during the rearing period, which sustainably affected the development and altered the metabolism. These changes could still be seen at first lactation. So, it is possible that, weaning age or mother’s parity will affect these animals in later life through metabolic imprinting. The existence and quality of long-term consequences are examined in an ongoing study with the same animals during their first and second lactation. Further research is needed to identify an optimal age to transition from MR to solid feed and an optimal amount of MR. It is challenging to distinguish the effect of older weaning age from the effect of an overall higher amount of MR that is consumed. Thus, it would be advisable to conduct further research on both factors and their influence on the growth and development of calves. Voluntary MR and solid feed intake are different in individual calves [26,45], and should therefore be considered in further research about optimal weaning age. From this study, a later weaning regimen can be considered as advantageous in early life with potential consequences for later health and metabolic performance. Naturally, calves suckling their dam were weaned at the age of 8–9 months [46,47]. This behavior might have been established through evolution, creating the best possible outcome for calves, and should therefore be considered in re-thinking weaning regimens in dairy calves.

## 5. Conclusions

Increasing weaning age to 17 weeks enables a smooth transition of physiological functions from the pseudomonogastric status to full ruminant status in dairy calves. However, weaning at 17 weeks of age is not only influenced by milk. The calves ingested up to 2 kg of concentrate feed, despite a high intake of MR. Thus, body maturation was supported by both sources of energy.

## Figures and Tables

**Table:** 

*p*-Values							
Parameters	Time (T)	Parity (P)	Weaning Age (W)	T × P	T × W	P × W	T × P × W
Live weight	**<0.001**	0.396	**<0.001**	0.685	**<0.001**	0.099	0.453
LWG	**<0.001**	0.422	**<0.001**	0.221	**<0.001**	0.325	0.556
Withers height	**<0.001**	0.419	**0.005**	0.444	**<0.001**	0.436	0.659
Hip height	**<0.001**	0.365	**0.002**	0.233	**<0.001**	0.364	0.985
Back length	**<0.001**	**0.035**	**0.008**	0.481	0.560	**0.011**	0.129
Heart girth	**<0.001**	0.122	**0.004**	0.899	**<0.001**	0.537	0.530
Body length	**<0.001**	0.791	**0.023**	0.140	**0.005**	0.116	0.217

**Table:** 

*p*-Values							
Parameters	Time (T)	Parity (P)	Weaning Age (W)	T × P	T × W	P × W	T × P × W
insulin	**<0.001**	0.654	0.089	0.790	**<0.001**	0.448	0.080
glucose	**<0.001**	0.224	**<0.001**	0.538	**<0.001**	0.620	0.781
total protein	**<0.001**	**0.021**	0.779	0.229	0.140	0.597	0.714
urea	**<0.001**	0.318	**<0.001**	0.064	**<0.001**	0.532	0.464

**Table:** 

*p*-Values							
Parameters	Time (T)	Parity (P)	Weaning Age (W)	T × P	T × W	P × W	T × P × W
cholesterol	**<0.001**	0.995	**<0.001**	0.990	**<0.001**	0.327	0.344
NEFA	**<0.001**	0.766	0.072	0.737	**<0.001**	0.735	0.440
BHB	**<0.001**	0.225	**0.026**	0.540	**<0.001**	0.685	0.511
leptin	**<0.001**	**0.025**	**<0.001**	0.448	**<0.05**	0.100	0.115

**Table 1:** Ingredients of milk replacer (MR) powder and concentrate feed.

Ingredients of MR Powder		Ingredients of Concentrate Feed	
**Component**	**g/kg**	**Component**	**g/kg**
Skimmed milk powder	320	Soybean meal	300
Sweet whey powder	198	Oat	305
Vegetable fat	140	Barley	180
Whey powder	102	Wheat	170
Whole milk powder	100	Soy bean oil	15
Buttermilk powder	100	Minerals and vitamins *	20
Minerals and vitamins	40	Calcium carbonate	10

* Ingredients per kg feed: 160 g Ca; 80 g P; 100 g Na; 30 g Mg; 1000 mg Fe; 800 mg Cu; 6000 mg Zn; 50 mg I; 50 mg Se; 30 mg Co; 800,000 IU vitamin A; 80,000 IU vitamin D3; 1000 mg vitamin E.

**Table 2:** Feeding regimen before and during experiment.

Experi-mental Day	Age in Days	MR Powder (g/L)		Available Volume of Liquid Feed per Day (L)		Available MR Powder per Day (kg)	
		Early Weaned	Late Weaned	Early Weaned	Late Weaned	Early Weaned	Late Weaned
	1–3 *	0	0	6	6	0	0
	3–5	150	150	6	6	Gradually increased from 0.3 to 0.9	Gradually increased from 0.3 to 0.9
	6 until start of experiment **	150	150	6	6	0.9	0.9
1 to 5		150	150	6	6	0.9	0.9
6 to 10		150	150	Gradually increased from 6 to 9	Gradually increased from 6 to 9	Gradually increased from 0.9 to 1.35	Gradually increased from 0.9 to 1.35
11 to 28		150	150	9	9	1.35	1.35
29 to 42		150	150	Gradually decreased from 9 to 2	9	Gradually decreased from 1.35 to 0.3	1.35
42 to 98		0	150	0	9	0	1.35
99 to 112		0	150	0	Gradually decreased from 9 to 2	0	Gradually decreased from 1.35 to 0.3
113 to 140		0	0	0	0	0	0

* 3 L of colostrum within 2 h after birth; pooled herd milk in the first three days of life. ** start of experiment at mean age of 8 ± 1.9 days, ranging from 6–12 days, one animal of earlyMC was 18 days old when entering the experiment.

**Table 3:** Ingredients of concentrate feed, roughage, milk replacer and total mixed ration (TMR).

Feed	DM %	XA g/kg T	XP g/kg T	XL g/kg T	XF g/kg T	NDF g/kg T	ADF g/kg T	Starch g/kg T	Sugar g/kg T
concentrate feed	86.84	63.21	232.31	47.64	68.63	199.09	82.61	363.67	48.32
roughage	86.06	66.61	98.28	22.05	326.74	660.53	365.18		
milk replacer	96.98	79.12	248.56	182.63					
TMR	39.29	43.75	81.22	35.35	217.76	426.36	244.81	301.51	

All ingredients were assessed by Weender analysis. Dry matter (DM), crude ash (XA), crude protein (XP) and crude fat (XL) were analyzed in all feedstuff. Crude fiber (XF), neutral detergent fiber (NDF) and acid detergent fiber (ADF) were analyzed in the solid feed. In concentrate feed and TMR starch was analyzed and in concentrate feed additionally sugar was analyzed.

**Table 4:** Morphometry of calves.

Measure	Definition
Withers height	From floor to dorsal process of first thoracic vertebra
Hip height	From floor to sacrum
Back length	From dorsal process of first thoracic vertebra to sacrum
Body length	From shoulder joint to ischium
Heart girth	Behind front leg

Definition of morphometry.
