# Correction: Schwarzkopf et al. Weaning Holstein Calves at 17 Weeks of Age Enables Smooth Transition from Liquid to Solid Feed. *Animals* 2019, *9*, 1132

**DOI:** 10.3390/ani11113044

**Published:** 2021-10-25

**Authors:** Sarah Schwarzkopf, Asako Kinoshita, Jeannette Kluess, Susanne Kersten, Ulrich Meyer, Korinna Huber, Sven Dänicke, Jana Frahm

**Affiliations:** 1 Institute of Animal Science, Faculty of Agricultural Sciences, University of Hohenheim, 70599 Stuttgart, Germany; sarah.schwarzkopf@uni-hohenheim.de (S.S.); asakokinoshita@googlemail.com (A.K.); korinna.huber@uni-hohenheim.de (K.H.); 2 Institute of Animal Nutrition, Friedrich-Loeffler-Institute, 38116 Braunschweig, Germany; jeannette.kluess@fli.de (J.K.); Susanne.kersten@fli.de (S.K.); ulrich.meyer@fli.de (U.M.); sven.daenicke@fli.de (S.D.)

The authors wish to make the following correction to their paper [1]. In the Materials and Method Section, Table 3 was incorrect due to some numerical errors. The correct version of the table appears below. The authors apologize for any inconvenience; the change does not affect the scientific results on the animal level.

All ingredients were assessed by Weender analysis. Dry matter (DM), crude ash (CA), crude protein (CP) and ether extract (EE) were analyzed in all feedstuff. Crude fiber (CF), neutral detergent fiber (NDF) and acid detergent fiber (ADF) were analyzed in the solid feed. In concentrate feed and TMR starch was analyzed and in concentrate feed additionally sugar was analyzed. 

## Figures and Tables

**Table 3:** Dry matter content and chemical composition of milk replacer, concentrate feed and roughage (hay) fed prior to weaning and total mixed ration (TMR) fed after weaning of the calves.

Feed	DM %	CA g/kg DM	CP g/kg DM	EE g/kg DM	CF g/kg DM	NDF g/kg DM	ADF g/kg DM	Starch g/kg DM	Sugar g/kg DM
**Prior to weaning**									
milk replacer	97	79	225	184					
concentrate feed	87	63	230	51	63	188	77	371	47
roughage (hay)	88	75	97	18	322	666	379		
**After weaning**									
TMR	44	73	134	36	212	421	242	182	12

## References

[1] Schwarzkopf S., Kinoshita A., Kluess J., Kersten S., Meyer U., Huber K., Dänicke S., Frahm J. (2019). Weaning Holstein Calves at 17 Weeks of Age Enables Smooth Transition from Liquid to Solid Feed. Animals.

